# Acute small-bowel obstruction caused by coiling of a ventriculoperitoneal shunt catheter: a case report

**DOI:** 10.1093/jscr/rjag1037

**Published:** 2026-11-10

**Authors:** Tarig H Alsanjak, Omer A Mohamed, Omer H Ibrahim, Alawad Y Abbas

**Affiliations:** Surgery Department, Almak Nimer University Hospital, Shendi University, Shendi, Sq7, 0000, Sudan; Surgery Department, Almak Nimer University Hospital, Shendi University, Shendi, Sq7, 0000, Sudan; Surgery Department, Almak Nimer University Hospital, Shendi University, Shendi, Sq7, 0000, Sudan; Surgery Department, Almak Nimer University Hospital, Shendi University, Shendi, Sq7, 0000, Sudan

**Keywords:** ventriculoperitoneal shunt, small-bowel obstruction, catheter coiling, acute abdomen, shunt complication

## Abstract

Ventriculoperitoneal (VP) shunting is widely used for the treatment of hydrocephalus, but abdominal complications can occasionally cause significant morbidity. We report a rare case of acute small-bowel obstruction caused by coiling of a distal VP shunt catheter around the ileum. A 65-year-old man with diabetes mellitus and hypertension presented with a 2-day history of abdominal pain, vomiting, progressive abdominal distension, and hiccups, three weeks after VP shunt placement for hydrocephalus. Abdominal radiography demonstrated features of mechanical small-bowel obstruction with an abnormal configuration of the distal shunt catheter. Emergency exploratory laparotomy revealed marked proximal small-bowel dilatation and the distal catheter coiled around the mid-ileum, producing a mechanical transition point. The bowel remained viable without ischaemia, necrosis, or perforation. The catheter was released and shortened, and no bowel resection was required. Early recognition of shunt-related intestinal obstruction and prompt surgical intervention may prevent irreversible bowel injury.

## Introduction

Ventriculoperitoneal (VP) shunting is one of the most commonly performed procedures for the treatment of hydrocephalus. Despite its effectiveness, VP shunts are associated with complications involving the central nervous system, shunt system, and abdominal cavity, including infection, obstruction, disconnection, migration, bowel perforation, pseudocyst formation, adhesions, catheter knotting, and intestinal obstruction [1–3].

The distal catheter may migrate, coil, knot, or become adherent to intra-abdominal structures, occasionally resulting in mechanical bowel obstruction. Reported mechanisms include catheter knotting, coiling around bowel loops, adhesions, and direct mechanical entanglement [3, 4]. Although uncommon, these complications can result in bowel ischaemia, necrosis, or perforation if diagnosis and treatment are delayed [4–6].

We present a case of acute small-bowel obstruction caused by coiling of the distal VP shunt catheter around the mid-ileum, successfully managed by emergency laparotomy with release and shortening of the catheter without bowel resection.

## Case report

A 65-year-old man with a 20-year history of diabetes mellitus and hypertension underwent VP shunt placement for benign hydrocephalus three weeks before presentation. He presented to the emergency department with a 2-day history of abdominal pain, vomiting, progressive abdominal distension, and hiccups. Clinical examination and plain abdominal radiography were consistent with small-bowel obstruction (Fig. 1). Laboratory investigations demonstrated leukocytosis with neutrophilia and mild hypokalaemia.

**Figure 1:**
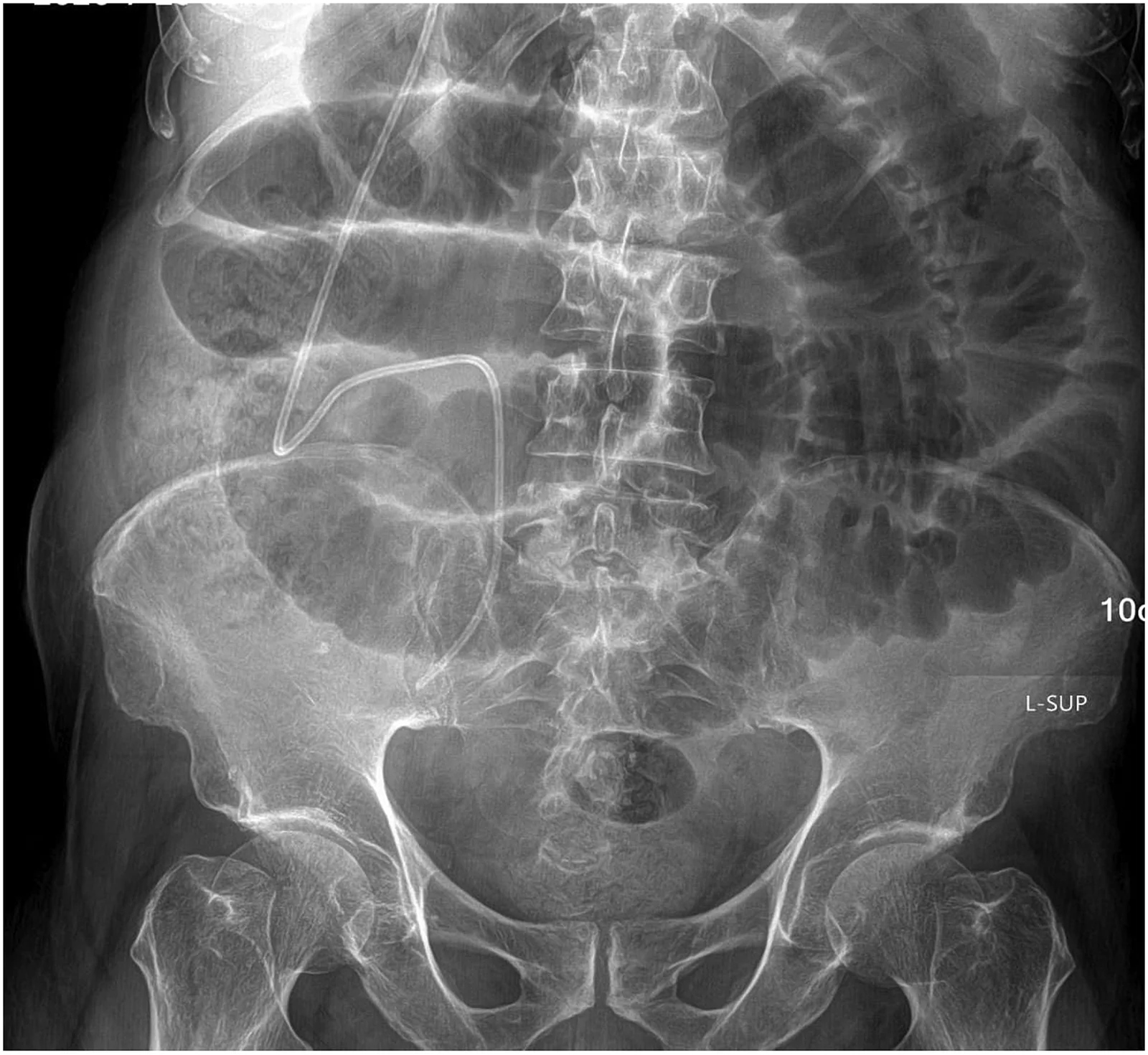
Preoperative abdominal radiograph. Plain abdominal radiograph demonstrating multiple dilated small-bowel loops with features consistent with mechanical small-bowel obstruction. The distal ventriculoperitoneal shunt catheter is visualized within the abdominal cavity, with its abnormal configuration raising suspicion of a shunt-related mechanical complication.

Given the clinical and radiological evidence of mechanical small-bowel obstruction, emergency exploratory laparotomy was performed. Intraoperatively (Fig. 2), the proximal small bowel was markedly dilated up to the mid-ileum, where the distal VP shunt catheter was found coiled around the bowel, producing a clear mechanical transition point. The bowel distal to the obstruction was collapsed. The involved bowel remained viable, with no evidence of ischaemia, necrosis, or perforation. The catheter was carefully released from the bowel, and the intraperitoneal portion was shortened. No bowel resection was required.

**Figure 2:**
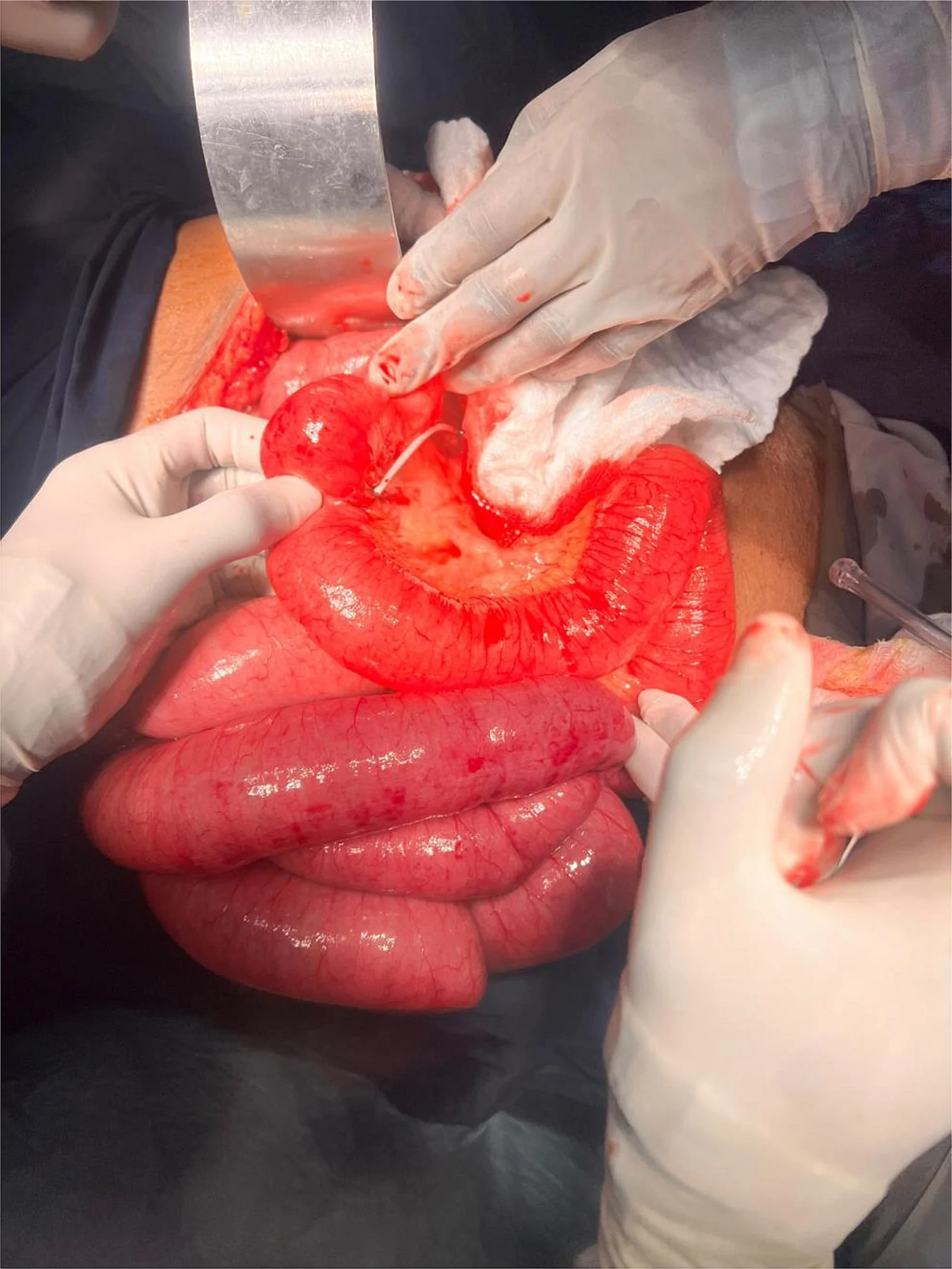
Intraoperative findings. Intraoperative photograph demonstrating the distal ventriculoperitoneal shunt catheter coiled around the mid-ileum, producing a mechanical transition point. The proximal small bowel is markedly dilated, while the distal bowel is collapsed. The involved bowel remains viable, with no evidence of ischaemia, necrosis, or perforation.

The postoperative course was uncomplicated, with recovery of bowel function and progressive clinical improvement. The patient was subsequently discharged home in stable condition.

## Discussion

Ventriculoperitoneal shunting is a widely used treatment for hydrocephalus, but complications may occur along the shunt system, including mechanical obstruction, migration, infection, and abdominal complications [3]. Although intra-abdominal complications are uncommon, they may result in serious morbidity, including intestinal obstruction, bowel ischaemia, necrosis, and perforation [6].

Imaging is essential for evaluating suspected abdominal shunt complications.

Abdominal radiography may show bowel obstruction and abnormal catheter configuration. Computed tomography provides detailed assessment of catheter position and its intra-abdominal relationships [7]. In the present case, abdominal radiography demonstrated features of mechanical small-bowel obstruction together with an abnormal configuration of the distal VP catheter, supporting the diagnosis and prompting emergency surgical exploration.

Intestinal obstruction caused by a VP shunt catheter is particularly uncommon, and several mechanisms have been reported, including catheter coiling, spontaneous knot formation, adhesions, and migration [1, 2, 4, 8, 9] The published cases summarized in Table 1 demonstrate a spectrum of VP shunt-related intestinal obstruction, ranging from reversible mechanical obstruction to bowel ischaemia and necrosis. Our case is comparable to the reports in which early release of the catheter restored bowel viability without requiring resection [1, 2], highlighting the importance of prompt recognition and surgical intervention.

**Table 1:** Selected published cases of intestinal obstruction associated with ventriculoperitoneal shunt catheters.

References	Year	Patient age (year)	Mechanism	Management
AlJoaid *et al.* [1]	2023	21	Closed-loop bowel obstruction caused by VP shunt catheter coiling at the sigmoid colon	Emergency laparotomy; the coiled catheter was identified and released from the sigmoid colon, and the distal catheter was shortened. No bowel resection was required
Laxman *et al.* [2]	2022	18	Bowel obstruction secondary to spontaneous knot formation of the VP shunt catheter	Exploratory laparoscopy; the knotted catheter was cut with laparoscopic scissors and removed, releasing the constriction. The bowel regained colour and viability, so no bowel resection was required
Xue *et al.* [8]	2022	65	Adhesive-band strangulation associated with the VP shunt catheter	Emergency laparotomy; adhesiolysis and partial resection of necrotic small bowel with side-to-side anastomosis. The intra-abdominal catheter was externalized
De Jesus and Rios-Vicil [4]	2021	25	Bowel obstruction and necrosis caused by VP shunt catheter knotting	Emergency exploratory laparotomy with release of the knotted catheter and resection of ~21 cm of necrotic small bowel, followed by restoration of bowel continuity
Joy *et al.* [9]	2020	Paediatric	Ischemic bowel secondary to VP shunt knotting	Urgent surgical exploration with release/removal of the knotted catheter and management of the ischemic bowel. The original report should be consulted for the exact operative details before specifying bowel resection in our table
Present case	2026	65	Acute intestinal obstruction caused by VP shunt catheter; no ischemia, necrosis, or perforation	Emergency exploratory laparotomy; the catheter was carefully released from the bowel and the intraperitoneal portion shortened. The bowel was viable, with no ischemia, necrosis or perforation; no bowel resection was required

In contrast, delayed or more severe obstruction may result in irreversible bowel injury. Xue *et al.* [8] reported adhesive-band strangulation associated with a VP catheter in a 65-year-old woman, requiring partial resection of necrotic small bowel and catheter externalization. De Jesus and Rios-Vicil [4] described a 25-year-old man with a tightly knotted catheter around the small bowel, resulting in necrosis and requiring resection of 21 cm of bowel. Joy *et al.* [9] similarly reported a paediatric patient with catheter knotting complicated by small-bowel strangulation, ischaemia, and necrosis. These cases demonstrate that the same underlying mechanical phenomenon can range from reversible obstruction to bowel necrosis, depending on the degree and duration of vascular compromise.

Our case is therefore notable for the combination of acute mechanical small-bowel obstruction, direct coiling of the distal catheter around the mid-ileum, and preservation of bowel viability despite the presence of a clear mechanical transition point. Unlike several previously reported cases complicated by bowel ischaemia or necrosis, our patient underwent early surgical exploration before irreversible bowel injury developed. The catheter was released and shortened, and no bowel resection was required.

The clinical presentation of VP shunt-related intestinal obstruction is nonspecific and may resemble other causes of acute abdomen. Abdominal pain, distension, vomiting, and other features of intestinal obstruction may occur. Therefore, a history of VP shunt placement should be actively sought in patients presenting with acute abdominal symptoms. An abnormal catheter configuration on imaging should further increase suspicion of a shunt-related mechanical complication.

Management should be guided by the mechanism of obstruction and the condition of the bowel. When the bowel is viable, release of the obstructing catheter and appropriate management of the distal shunt may be sufficient [1, 2]. When strangulation has resulted in ischaemia, necrosis, or perforation, bowel resection may be required [4, 8, 9]. The literature therefore supports early surgical assessment when a VP catheter is implicated in mechanical bowel obstruction.

## Conclusion

Ventriculoperitoneal shunt catheter-related small-bowel obstruction is a rare but potentially serious abdominal complication. Catheter coiling around the bowel can produce acute mechanical obstruction and may progress to ischaemia, necrosis, or perforation if diagnosis is delayed. A history of VP shunt placement should therefore be considered in patients presenting with acute intestinal obstruction or unexplained abdominal symptoms. Early imaging, recognition of an abnormal catheter configuration, and timely surgical intervention may permit release of the obstruction before irreversible bowel injury develops and potentially avoid bowel resection.
